# Serum copeptin and neuron specific enolase are markers of neonatal distress and long-term neurodevelopmental outcome

**DOI:** 10.1371/journal.pone.0184593

**Published:** 2017-09-20

**Authors:** Dorottya Kelen, Csilla Andorka, Miklós Szabó, Aleksander Alafuzoff, Kai Kaila, Milla Summanen

**Affiliations:** 1 First Department of Pediatrics, Semmelweis University, Budapest, Hungary; 2 Department of Biosciences, University of Helsinki, Helsinki, Finland; 3 Neuroscience Center, University of Helsinki, Helsinki, Finland; Univesity of Iowa, UNITED STATES

## Abstract

The objective of this study was to evaluate the early changes in serial serum levels of copeptin and neuron-specific enolase (NSE) in neonates diagnosed with birth asphyxia, and to determine whether these biomarkers measured in the first 168 hours after birth are predictive of long-term neurodevelopmental outcome. Copeptin and NSE levels were measured from serum samples collected 6, 12, 24, 48, 72, and 168 hours after birth from 75 term neonates diagnosed with hypoxic-ischemic encephalopathy (HIE) and treated with therapeutic hypothermia for 72 hours. In addition, serum copeptin levels after birth were measured from 10 HIE diagnosed neonates, who were randomized to the normothermic arm of the TOBY cohort. All neonates underwent neurodevelopmental assessment using the Bayley Scales of Infant and Toddler Development-II at two years of age. Copeptin levels were highest at 6 hours after birth and steadily decreased, whereas the highest NSE levels were measured at 24 hours after birth. The biomarker levels correlated with blood-gas parameters (base excess, pH and lactate) at 6 and 12 hours after birth. Copeptin and NSE levels in the early postnatal period were significantly higher in neonates with poor outcome compared to those with favorable outcome at two years of age. Furthermore, in the TOBY cohort, copeptin levels were significantly lower in hypothermic compared to normothermic neonates. To conclude, copeptin and NSE measured in the early postnatal period are potential prognostic biomarkers of long-term neurodevelopmental outcome in term neonates diagnosed with HIE and treated with therapeutic hypothermia.

## Introduction

Hypoxic-ischemic encephalopathy (HIE) is a significant cause of mortality and morbidity, occurring in 1.5 out of 1000 infants in developed countries [1]. Therapeutic hypothermia was shown to improve neurological outcome, and is now the standard method of care for infants with HIE [2]. However, at least half of these infants still have an unfavorable outcome, and putative adjuvant therapies are widely studied [3]. There is pressing demand for reliable biomarkers to guide early clinical decisions, and to help counsel parents. Useful and reliable biomarkers of outcome after HIE would need to have high specificity and sensitivity, to be of use in clinical settings, and to be readily available for fast assays within the time window of therapeutic decisions.

Studies focusing on early biomarkers are being carried out using a wide range of approaches. For instance, magnetic resonance imaging (MRI) is a promising tool to predict outcome, but its availability is limited [4]. Moreover, the timing of an MRI scan usually exceeds the immediate postnatal period. Amplitude-integrated EEG is also used for prognosis, but its predictive value is modest [2]. As cerebrospinal fluid samples are not feasible for routine use, and saliva or urine samples have not been widely studied, serum samples remain the most reasonable source of putative novel biomarkers of neonatal HIE. Several prognostic biomarkers of HIE have already been investigated and, in light of available data [5–7], neuron-specific enolase (NSE), a glycolytic enzyme from neurons and neuroendocrine cells, appears promising.

In this study, we have investigated copeptin, the biochemically stable C-terminal fragment of prepro-arginine vasopressin (AVP), as a novel biomarker to predict long-term neurodevelopmental outcome after birth asphyxia, with parallel comparative assays of NSE. Copeptin has been found to be predictive in several conditions affecting the brain, such as stroke and traumatic brain injury [8–10]. Notably, AVP is released from the pituitary to the circulation following activation of the hypothalamic-pituitary-adrenal axis (HPA-axis) in response to stressful stimuli [11]. In line with the pronounced fetal endocrine activation at birth, extremely high copeptin concentrations in the neonate blood have been found after vaginal birth [12], and copeptin levels are further elevated following birth asphyxia [13,14]. The specific aim of this work was to study the levels of, and relationship between, NSE and copeptin concentrations as well as blood acid-base parameters in the early postnatal hours in infants with HIE treated with therapeutic hypothermia, and to examine the data in relation to neurodevelopmental outcome at two years of age. The material in this study also permitted examining the effect of therapeutic hypothermia on serum levels of copeptin.

## Methods

### Study design and patients

The study was conducted at a tertiary neonatal unit of the 1^st^ Department of Pediatrics at Semmelweis University, Budapest, Hungary. Ethical approval was obtained from the Hungarian National Ethics Committee for Medical Research (TUKEB 591/KO/2004 and TUKEB 11790-2/2016/EKU 0235/16). Informed consent was obtained from all parents of the neonates who were enrolled in the study. 110 term neonates diagnosed with birth asphyxia born between 2005 and 2011 were included in the study. Of the 110 neonates, 35 were excluded due to no or shortened period of cooling (n = 22), congenital malformation (n = 1), sudden infant death syndrome (n = 1), subdural bleed (n = 1), intrauterine growth restriction (n = 1), prematurity (n = 4), and no follow-up (n = 5), leaving 75 neonates for the final analysis. Clinical criteria for diagnosing moderate-to-severe HIE, and treatment with moderate whole-body cooling were defined as follows: (i) at least one of the following: continued need for ventilation at 10 mins after birth; 5 minute Apgar score ≤ 5; pH < 7.0 or base excess (BE) ≤ -16 mmol/L within 60 min after birth, and (ii) altered level of consciousness, and at least one of the following: hypotonia, abnormal reflexes, or seizures [15]. Blood samples for the diagnosis were obtained within 60 minutes after birth.

### Clinical care

Standard intensive care and therapeutic hypothermia were provided as described before [6,15–17]. In neonates treated with hypothermia, cooling was induced within 6 hours of birth and the target temperature (33–34°C), monitored continuously by rectal probe, was reached by a mean of 4 hours of age. Therapeutic hypothermia was maintained for 72 hours, and rewarming was done at a rate of 0.5°C/hour until normal rectal temperature (37°C ± 0.5) was reached. For the analyses on the effect of cooling on copeptin levels we used data from an additional 10 normothermic neonates from 2005–2006 who were randomized to the non-cooled group in the international TOBY trial [15]. These 10 samples have been analyzed for NSE concentrations already previously [6]. Standard intensive care was provided as above and rectal temperature was maintained at 37 ± 0.2°C in these neonates allocated to normothermia.

### Copeptin, NSE and blood gas analyses

Blood gas measurements were taken as clinically indicated, and blood samples were analyzed with the Abbott i-STAT^®^1 blood gas analyzer. pH and lactate values were corrected for temperature during the analysis, and BE values were derived from these parameters.

Serum samples for biomarker analysis from each of the 110 patients were collected at least at one of the following time points: 6, 12, 24, 48, 72 and 168 hours of age, by taking an extra 400 μl of venous blood in addition to the blood sampling for standard investigations. Blood samples were then centrifuged/separated, and all of the serum samples, including those from the TOBY trial, were immediately frozen and kept at -80°C until analysis. On average, four samples per patient were collected and analyzed. In total, 453 and 452 serum samples were analyzed for copeptin and NSE, respectively. Copeptin and NSE concentrations were measured by the B.R.A.H.M.S. KRYPTOR Compact PLUS (Thermo Scientific) in November 2015. Considering the remarkable biochemical stability of copeptin [11], we have no reason to assume that the biomarker concentrations would be significantly affected by long-term storage at -80°C. Furthermore, since all of the samples used in this study were equally old at the time of analysis, possible storage effects would be similar for all samples.

The detection limits for copeptin and NSE were 0.9 pmol/l and 0.8 ng/ml, respectively. For copeptin, the functional sensitivity (20% CV) was assessed as < 2 pmol/l, and the intra- and inter-assay CVs for copeptin concentrations > 15 pmol/l were < 4% and < 5%, respectively. For NSE, the functional sensitivity (20% CV) was assessed as 4.4 ng/ml, and the intra- and inter-assay CVs were 9.5% and 15.3%, respectively.

### Perinatal parameters

Clinical information about mode of delivery, birth characteristics of the infants (gestational age, gender, birthweight, Apgar scores at 5 and 10 min, temperature data), and postnatal acid-base parameters were collected from hospital charts and from the electronic institutional database.

### Two-year follow-up

Fourteen of the studied neonates died during the neonatal period (1–27 days). Neurodevelopmental assessment using the Bayley Scales of Infant and Toddler Development TM II [18] was performed for the remaining neonates between 17 and 28 months of age (mean 21.4 ± 2.6) by examiners who were blinded to clinical history and biomarker results. Mental Developmental Index (MDI) and Psychomotor Developmental Index (PDI) scores were calculated, and the neonates were classified into two groups: favorable outcome (survival without severe disability; MDI and PDI ≥ 70), and poor outcome (severe disability; MDI or PDI <70, or death).

### Statistical analysis

Statistical analyses were performed with GraphPad Prism 6 or SPSS 22. Correlations between the pH, BE, lactate, copeptin and NSE levels were calculated with Spearman correlation coefficient. Copeptin and NSE concentrations between time points were compared with the Kruskal-Wallis test and Dunn’s multiple comparisons test. Copeptin and NSE concentrations between the study groups were compared using the Mann-Whitney U test. Fisher’s exact test was used to determine significant differences in the study population. A logistic regression model was fit to the binary outcome using copeptin and NSE concentrations as predictors. Receiver operating characteristic (ROC) analysis was used to evaluate the predictive accuracy of the logistic regression model, as well as copeptin and NSE separately. *P*-values below 0.05 were considered statistically significant, except for multiple Mann-Whitney U test comparisons, where the Holm-Sidak method was used to compute thresholds to ensure a family-wise error rate below 0.05. Statistical significance was denoted with * when p ≤ 0.05, with ** when p ≤ 0.01, and *** when p ≤ 0.001.

## Results

### Copeptin and NSE levels after birth

**Fig 1:**
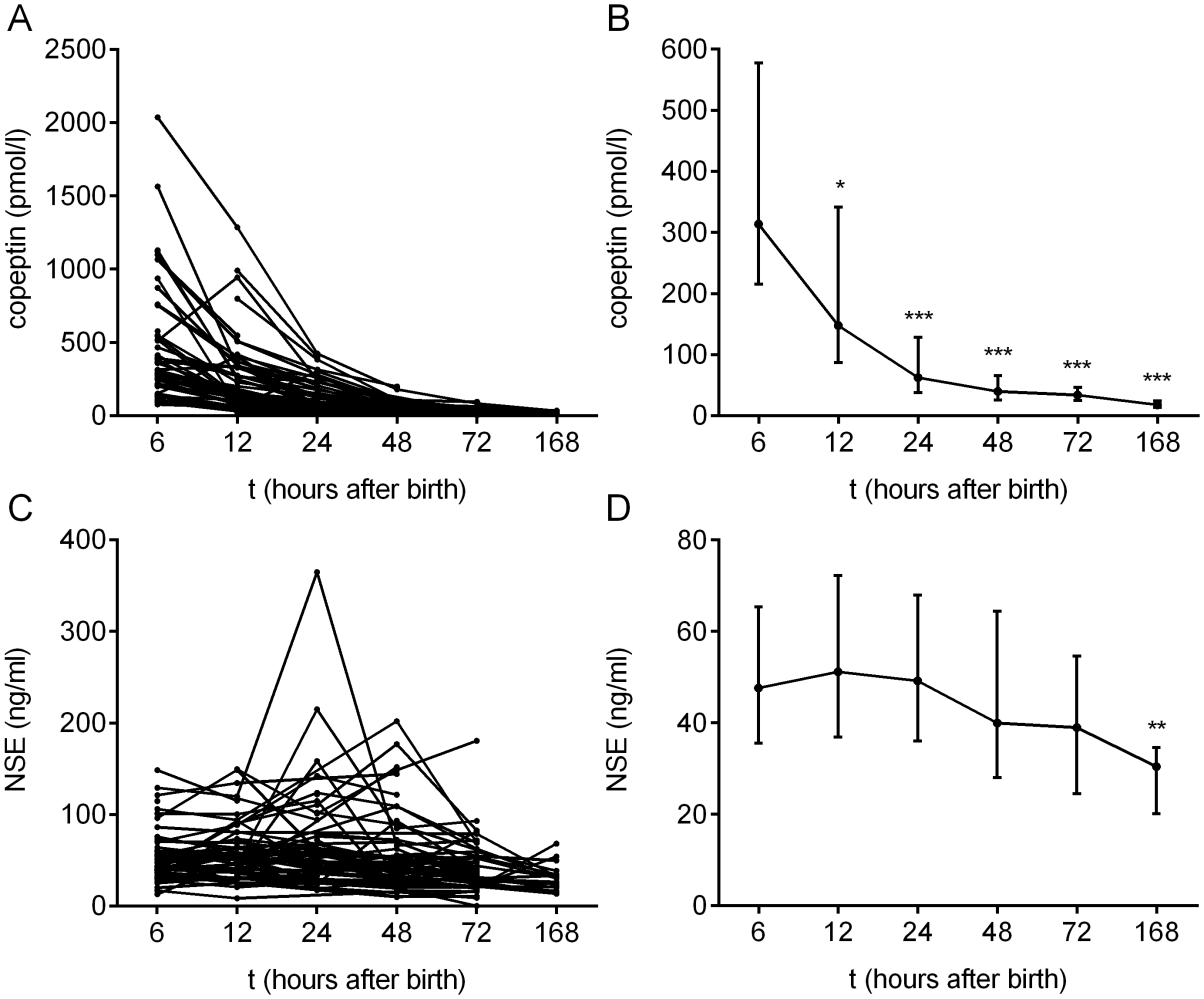
Copeptin and NSE concentrations in serum samples over time (n = 75). (A, B) Copeptin levels decrease over time, with highest levels measured at 6 hours after birth. Copeptin concentrations are shown for individual neonates (A) as well as the median ±IQR concentration for each time point (B). Compared to the 6 hour time point, concentrations were significantly lower at all subsequent time points (p = 0.0448 at 6 vs. 12 hours, and p < 0.0001 at all other time points). (C, D) NSE levels are relatively stable until 24 h after birth, after which the levels decrease. Data is shown for individual neonates (C) and as the median ± IQR at each time point (D). Compared to the 6 hour time point, concentrations were significantly lower only at 168 hours after birth (p = 0.0013). The Kruskal-Wallis test with Dunn’s multiple comparisons test was used.

The copeptin and NSE concentrations in the individual neonates treated with therapeutic hypothermia (n = 75), as well as the median biomarker concentrations as a function of time, are shown in Fig 1. Copeptin concentrations were highest at six hours after birth (median 313.5 pmol/l, IQR 215.2–577.6 pmol/l) and steadily decreased over time. The concentrations at 12, 24, 48 and 72 hours after birth were significantly lower than what was measured at 6 hours (Fig 1B). NSE concentrations were relatively stable until 24 hours after birth (median at 24 hours was 49.2 ng/ml, IQR 36.0–68.0 ng/ml), and thereafter decreased to a median of 30.4 ng/ml at 168 hours after birth (IQR 20.1–34.6 ng/ml). When compared to the 6 hour time point, NSE concentrations were significantly lower only at 168 hours after birth (Fig 1D).

### Correlations of copeptin and NSE levels with blood gas parameters

Several previous studies have reported that copeptin concentrations in umbilical cord samples strongly correlate with umbilical blood pH and BE [12–14,19]. We observed here that copeptin and NSE concentrations at 6 and 12 hours after birth still correlate with the pH and BE, as well as lactate (S1 Fig). Those individuals with lower pH and BE, and higher lactate had higher copeptin and NSE levels both at 6 and 12 hours of life.

### Biomarkers and mode of delivery

Of the 75 neonates included in the study, 48 were born by vaginal delivery (7 deliveries assisted by vacuum extraction) and 27 by emergency cesarean section. The mode of delivery had no effect on copeptin levels at any of the studied time points (6–72 hours after birth). The NSE levels at 6 hours after birth were significantly higher in the neonates born via emergency cesarean section compared to vaginal delivery (medians 71.1 and 43.8 ng/ml respectively, p = 0.0404). There were no differences in NSE levels with respect to delivery mode at any of the other time points.

### Biomarker levels and two-year outcome

The 75 neonates were subjected to neurodevelopmental assessment using the Bayley Scales of Infant and Toddler Development TM II at around two years of age. The neonates were divided into two groups based on the MDI and PDI scores (see Methods): favorable outcome and poor outcome. The 14 neonates who died were included in the poor outcome group. The clinical characteristics of neonates in the two outcome groups are shown in Table 1. Gestational age, birthweight, gender, and time to reach target temperature were similar in the two groups. The Apgar scores at 5 and 10 minutes were significantly lower in the poor outcome group, and emergency cesarean section was more frequent in the poor outcome group (60% vs 24%, p = 0.0044).

**Table 1:** Clinical characteristics of the study groups.

		Favorable outcome (n = 50)	Poor outcome (n = 25)	P-value
Gestational age		39.5 (*38–40*)	39 (*37–40*)	0.2087
Birth weight (g)		3200 (*2860–3510*)	3200 (*2755–3400*)	0.4286
Male		28 (*56*)	17 (*68*)	0.4537
5 min Apgar score		4.5 (*3–6*)	3 (*2–4*.*75*)	0.0072
10 min Apgar score		6 (*5–7*)	4 (*2–4*.*75*)	0.0002
Target temperature reached (h)		4.02 (*2*.*02–5*.*80*)	2.92 (*1*.*77–5*.*54*)	0.2883
Emergency cesarean section		12 (*24*)	15 (*60*)	0.0044
Serum copeptin (pmol/l)	6 h	273.8 *(150*.*5–413*.*6)*	530.2 *(360*.*1–1067*.*0)*	0.0068
	12 h	124.1 *(68*.*4–275*.*1)*	269.8 *(160*.*9–518*.*3)*	0.0050
	24 h	57.9 *(31*.*3–94*.*1)*	108.4 *(45*.*7–243*.*3)*	0.0525
	48 h	32.3 *(23*.*0–53*.*4)*	65.3 *(40*.*3–76*.*0)*	0.0226
	72 h	31.6 *(23*.*8–43*.*6)*	44.2 *(30*.*0–53*.*9)*	0.0781
Serum NSE (ng/ml)	6 h	41.2 *(30*.*1–54*.*2)*	66.8 *(49*.*2–112*.*3)*	0.0009
	12 h	46.1 *(31*.*3–55*.*7)*	89.2 *(53*.*7–115*.*6)*	0.0005
	24 h	47.3 *(28*.*3–57*.*4)*	101.0 *(47*.*6–123*.*7)*	0.0005
	48 h	35.8 *(25*.*6–49*.*7)*	72.1 *(38*.*1–127*.*2)*	0.0009
	72 h	34.4 *(21*.*0–46*.*9)*	60.4 *(38*.*1–74*.*5)*	0.0029

Median (*interquartile range*) or number (*percentage*) are shown.

**Fig 2:**
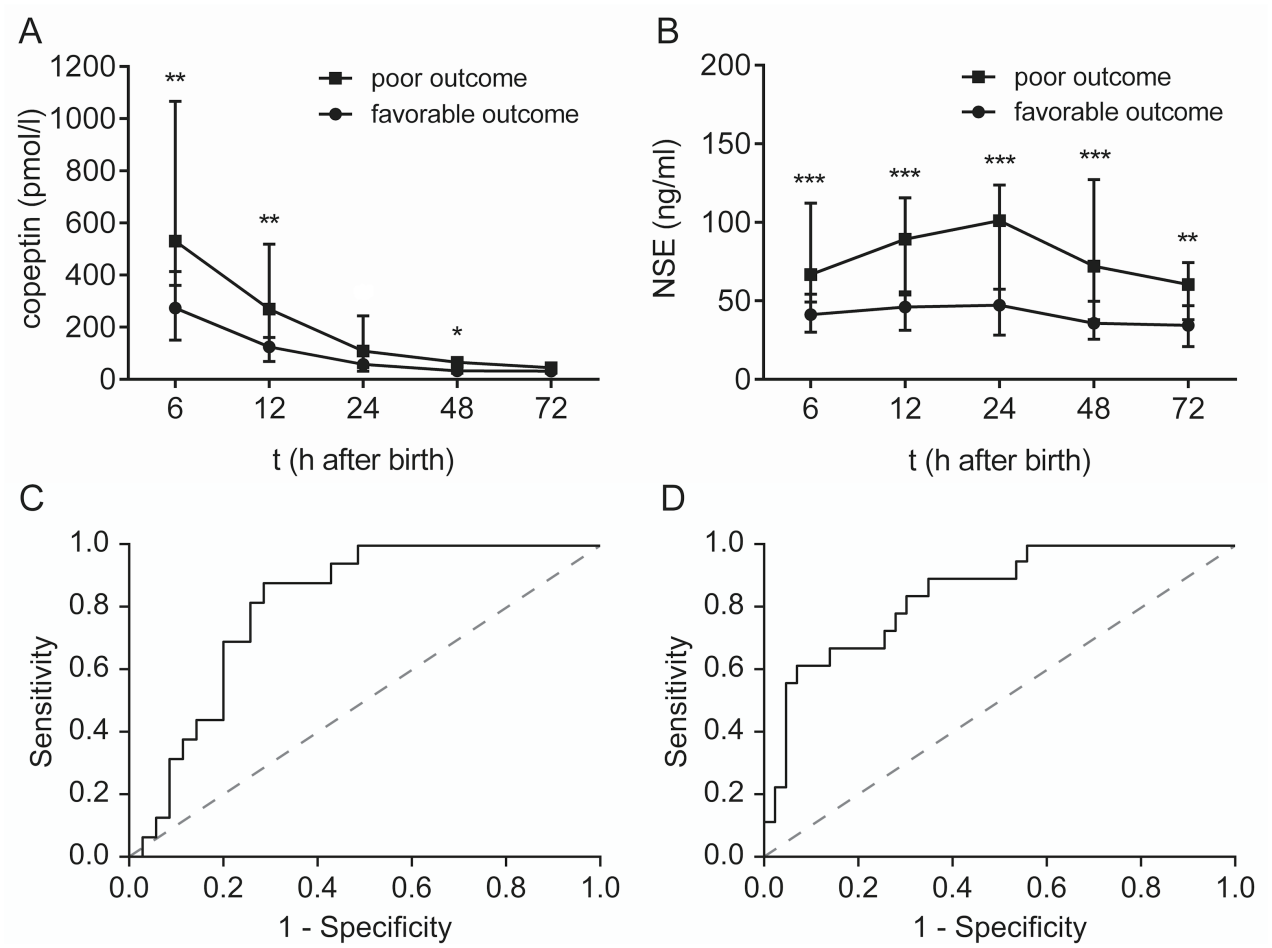
Copeptin and NSE levels after birth in relation to two-year outcome. (A) Copeptin levels were significantly higher in neonates in the poor outcome group (n = 25) than in the favorable outcome group (n = 50) at 6 hours (p = 0.0068), 12 hours (p = 0.0050) and 48 hours (p = 0.0226) after birth. (B) NSE levels were significantly higher in the neonates in the poor outcome group compared to the favorable outcome group at all time points (p = 0.0009 at 6 hours, p = 0.0005 at 12 hours, p = 0.0005 at 24 hours, p = 0.0009 at 48 hours, and p = 0.0029 at 72 hours). (C-D) ROC curves of combined copeptin and NSE concentrations at 6 hours (C) and 12 hours (D) after birth in relation to two year neurodevelopmental outcome. Graphs A and B show the median ± IQR. The data for each time point were analyzed separately with the Mann-Whitney U-test, and the Holm-Sidak method was used to compute thresholds to ensure a family-wise error rate below 0.05.

Both copeptin and NSE levels in the early postnatal period were significantly higher in the poor outcome group compared to the favorable outcome group (Fig 2A and 2B and Table 1). The difference in copeptin levels between the two outcome groups was significant at 6, 12 and 48 hours after birth. For NSE, significant differences were found at all time points studied (6–72 h). Interestingly, a significant correlation between copeptin and NSE serum concentrations was found at all time points studied (r = 0.2862, p = 0.0439 at 6 hours; r = 0.4744, p = 0.0001 at 12 hours; r = 0.3087, p = 0.0164 at 24 hours; r = 0.3710, p = 0.0032 at 48 hours; and r = 0.3097, p = 0.0286 at 72 hours after birth).

Strikingly, we found that ROC curve analyses of both copeptin and NSE concentrations at 6 and 12 hours after birth discriminated between favorable and poor outcome at two years of age. Moreover, ROC analysis on the combination of the two biomarkers showed an even higher level of accuracy. At 6 hours after birth, the area under the curve for copeptin was 0.770 (95% CI 0.64–0.90, p = 0.002), for NSE 0.786 (95% CI 0.65–0.92, p = 0.001) and for their combination 0.814 (95% CI 0.70–0.93, p < 0.001, Fig 2C). At 12 hours after birth, the corresponding values were 0.764 (95% CI 0.64–0.89, p = 0.001), 0.839 (95% CI 0.73–0.95, p < 0.001) and 0.848 (95% CI 0.75–0.95, p < 0.001, Fig 2D).

### Copeptin levels and therapeutic hypothermia

**Fig 3:**
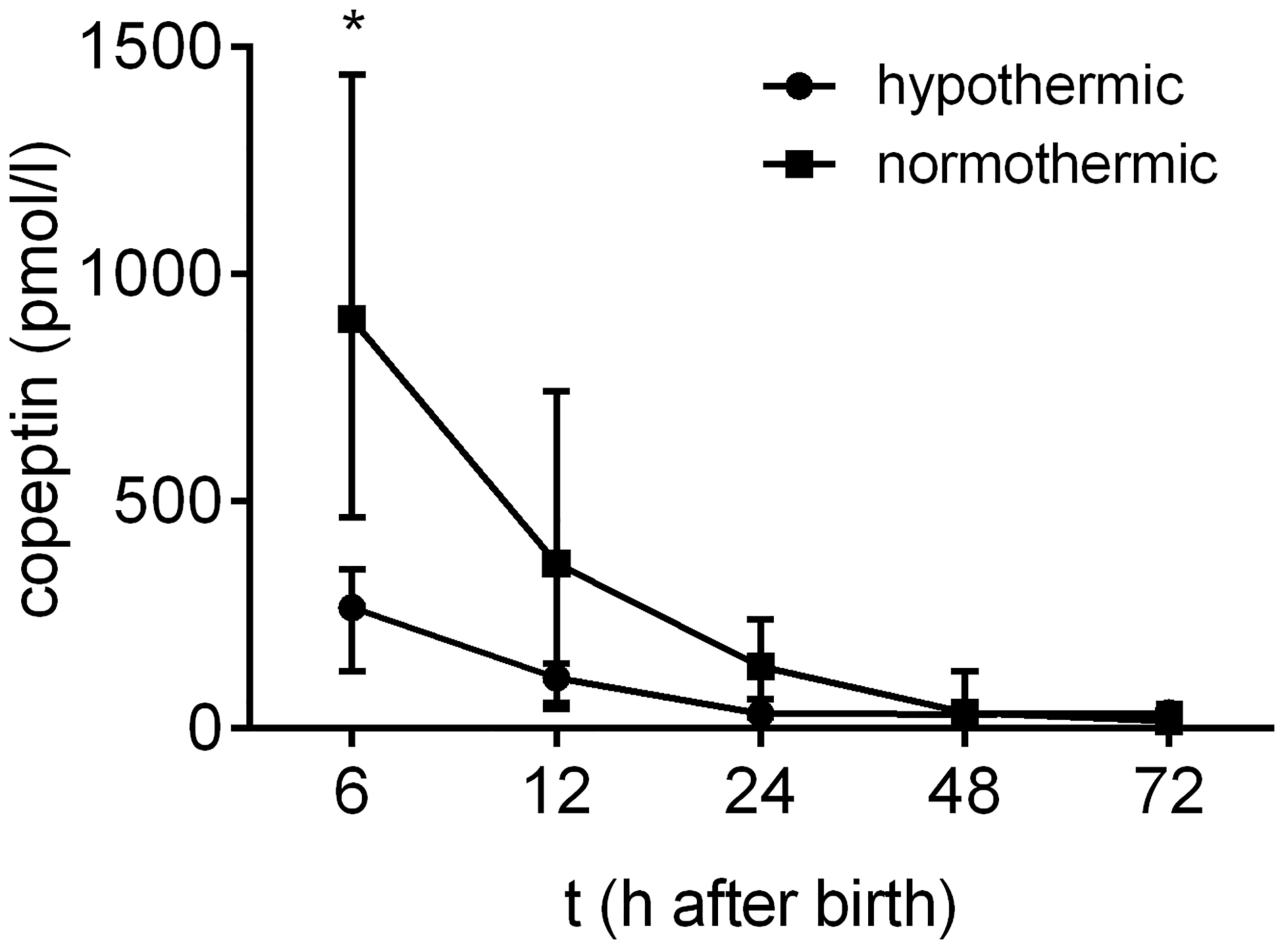
Copeptin levels in relation to therapeutic hypothermia. Copeptin concentrations after birth were lower in the hypothermic neonates from the TOBY cohort (n = 11) compared to the normothermic neonates from the same cohort (n = 10) at 6 hours after birth (p = 0.0495). Graph shows the median ± IQR. The data for each time point was analyzed separately with the Mann-Whitney U-test, and the Holm-Sidak method was used to compute thresholds to ensure a family-wise error rate below 0.05.

Some of the samples that were used in this study originated from the TOBY trial [15], which gave us the unique opportunity to compare copeptin concentrations in normothermic and hypothermic neonates diagnosed with HIE. Only neonates from the TOBY trial (10 normothermic and 11 hypothermic) were included in this analysis to ensure a valid comparison of the two groups. We have previously measured the NSE concentrations in these samples [6], and found no difference between normothermic and hypothermic neonates regarding their NSE levels. However, copeptin levels were significantly lower in the hypothermic neonates compared to the normothermic neonates of the TOBY cohort at 6 hours after birth (p = 0.0495, Fig 3). Out of the 11 hypothermic neonates of the TOBY cohort, 10 had favorable outcome and one developed poor outcome, whereas three out of the 10 normothermic neonates had poor outcome at two years of age.

## Discussion

There is a pressing need for the identification and characterization of readily accessible, and reliable biomarkers of birth asphyxia to facilitate clinical decisions during the first critical hours after birth. The main aim of this work was to test the utility of copeptin and NSE as prognostic biomarkers of HIE in a cohort of asphyxiated neonates treated with therapeutic hypothermia for 72 hours.

Our study was carried out in a tertiary care center with exclusively ex-utero infants, which precluded blood sampling immediately after birth, but the data obtained from the 6^th^ to the 168^th^ postnatal hour showed a progressive decline in the serum copeptin concentrations in the study population, indicating that the majority of AVP/copeptin is released at birth, but taking into account the approximately 30 minute half-life of copeptin [20], a significant amount of AVP/copeptin release is still ongoing during the hours and days after birth in these HIE-diagnosed neonates. In a study by Schlapbach *et al*. the median copeptin concentration in the umbilical cord blood serum of asphyxiated neonates was 919 pmol/l [13], and in the present study the median at six hours after birth 314 pmol/l, further demonstrating that the bulk AVP/copeptin release occurs at birth. In contrast to this, NSE levels remained relatively stable and they were comparable to those reported in other studies with asphyxiated neonates [5,6]. Notably, the serum levels of both biomarkers showed a robust, statistically significant correlation with blood-gas parameters (negative base excess, low pH, and high lactate) at 6 and 12 hours after birth.

The most important finding in the present study is that high levels of both copeptin and NSE are predictive of poor outcome at two years of age. Moreover, using the two biomarkers in combination showed an even higher level of accuracy in the ROC curve analysis. To our knowledge, this is the first study of copeptin and long-term neurodevelopmental outcome, while several previous studies have looked at the relationship between NSE levels and neurodevelopmental outcome [5,6,21,22], with promising but also some conflicting results. Nagdyman *et al*. [21] state that NSE concentrations did not supply reliable information about neurodevelopmental outcome after birth asphyxia, whereas other studies have reported that serum NSE levels have predictive capacity for poor outcome [5,6,22]. Considering the fact that both high copeptin and high NSE concentrations were predictive of poor neurodevelopmental outcome, it is no surprise that a significant correlation between copeptin and NSE levels was found at all time points studied.

The present study population, which included samples from the original TOBY trial [15], provided us also with the unique opportunity to examine the effects of therapeutic hypothermia on copeptin concentrations after birth. We have already shown [6] that there were no differences in NSE levels between these normothermic and hypothermic neonates. Interestingly, copeptin levels were significantly higher in normothermic neonates compared to hypothermic neonates at 6 hours after birth, indicating that therapeutic hypothermia decreases the peripheral release of copeptin. This is in agreement with the fact that AVP neurons are intrinsically thermosensitive and hypothermia seems to inhibit their activity [23–25]. Given the protective role of the fetal HPA-axis activation during birth [19,26], this raises the important question, to be analyzed in further studies, of whether the hypothermia induced decrease in AVP secretion is beneficial.

Although both biomarkers were predictive of poor long-term outcome in these neonates, we propose that copeptin is a more promising, clinically relevant biomarker for several reasons. First of all, the physiological mechanisms of fetal AVP release during birth, and birth asphyxia, are well understood [19]. A massive surge of AVP release takes place during normal vaginal birth in response to activation of the fetal HPA-axis, and this release is further accentuated by various kinds of stressors [12,26,27], including birth asphyxia [13,14]. Furthermore, in a very recent paper, Wellmann *et al*. showed that even a few contractions induced by exogenous oxytocin before primary cesarean section are enough to increase the copeptin concentrations in the umbilical cord serum of the neonate, indicating that AVP release is directly linked to transient periods of hypoxia [28]. Secondly, the time course of AVP/copeptin release, with highest levels measured in umbilical cord serum followed by a progressive decline in serum concentrations, is an advantageous property of an early biomarker, assisting in clinical decisions regarding treatment of asphyxiated neonates already in the first hours after birth. In contrast, in the present and in previous studies [6,21,22], NSE levels peaked several hours or even a day after birth, at which point initial clinical decisions have already been made. Finally, a practical advantage of copeptin is that it is already widely used as a biomarker of various pathophysiological states in adult patients [10], and therefore this approach can be easily adapted to neonatal intensive care units.

To conclude, in this study both copeptin and NSE were identified as promising prognostic biomarkers of neurodevelopmental outcome after HIE. The prognostic value of these biomarkers and their combination needs to be validated in a prospective, independent, large multicenter study, including umbilical cord serum samples and samples collected within the first 12 hours of life, which is a realistic time frame also in many tertiary neonatal units. Furthermore, future research should aim at analyzing the samples within a year of collection to exclude possible effects of long-term storage on biomarker concentrations. Using these serum biomarkers may turn out to be useful in the prediction of the long-term consequences of hypoxic ischemic insult, in assistance in clinical decision making, in selecting patients for further neuroprotective trials, in monitoring the progress of the disease, and in counseling the parents [29–31]. Based on the present and previous results [12–14], we propose that copeptin is a very promising candidate to fulfill the criteria of a prognostic biomarker for outcome after HIE.

## Supporting information

S1 Fig Biomarker correlations with blood gas parameters. Correlations of copeptin at 6 h (row 1; A-C), copeptin at 12 h (row 2; D-F), NSE at 6 h (row 3; G-I) and NSE at 12 h (row 4; J-L) with blood pH (column 1; A, D, G and J), blood base excess (column 2; B, E, H and K) and blood lactate (column 3; C, F, I and L). All correlations were significant, r- and p-values are shown in each panel.
(TIF)
