# Supplementary figures and images for: Serum copeptin and neuron specific enolase are markers of neonatal distress and long-term neurodevelopmental outcome
Source: PLoS One. 2017 Sep 20;12(9):e0184593. doi: 10.1371/journal.pone.0184593 (PMC5607206; doi:10.1371/journal.pone.0184593)

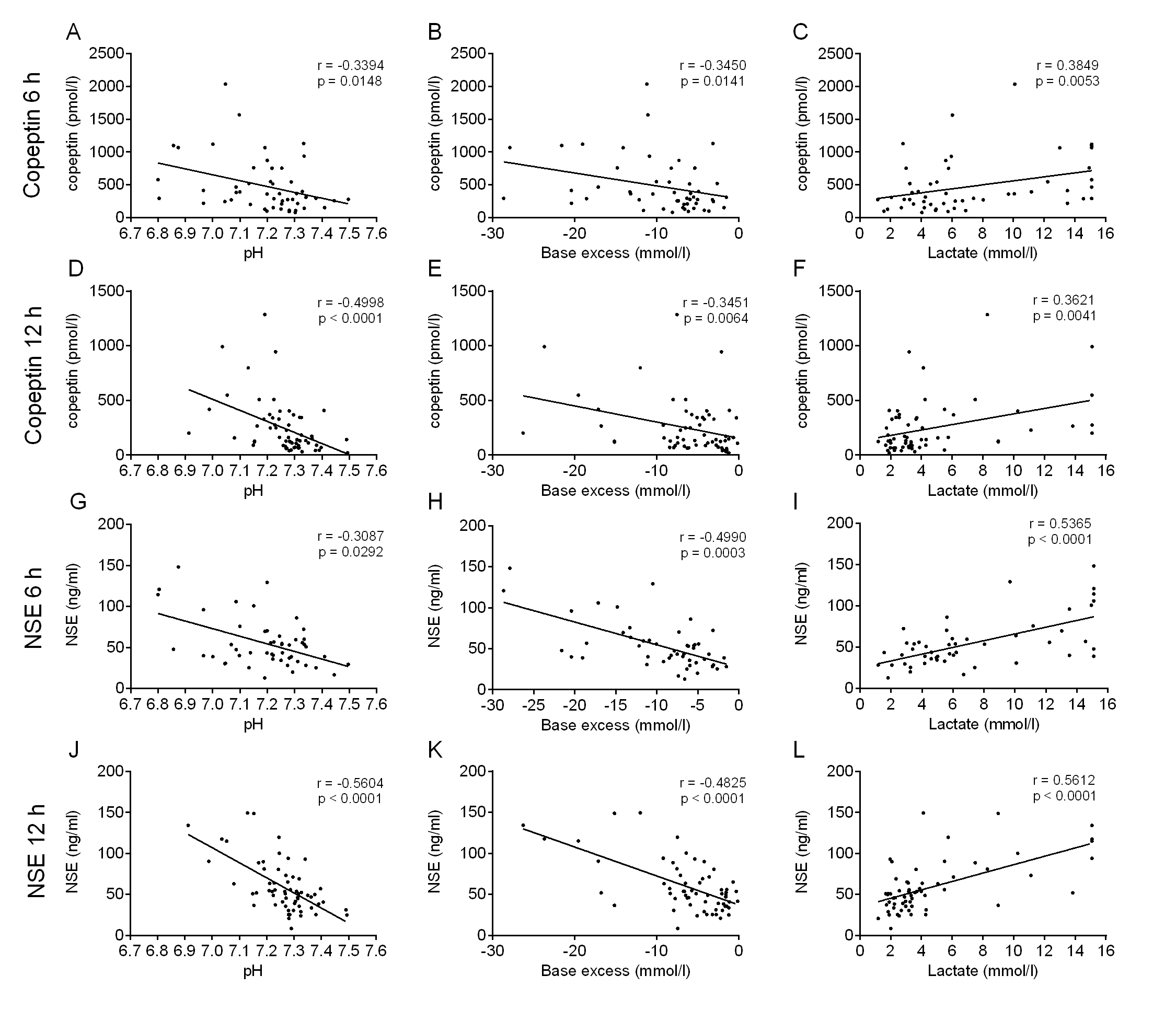

Supplement: S1 Fig — Correlations of copeptin at 6 h (row 1; A-C), copeptin at 12 h (row 2; D-F), NSE at 6 h (row 3; G-I) and NSE at 12 h (row 4; J-L) with blood pH (column 1; A, D, G and J), blood base excess (column 2; B, E, H and K) and blood lactate (column 3; C, F, I and L). All correlations were significant, r- and p-values are shown in each panel. (TIF) [file pone.0184593.s001.tif]
